# Internet-Related Instruments (Bergen Social Media Addiction Scale, Smartphone Application-Based Addiction Scale, Internet Gaming Disorder Scale-Short Form, and Nomophobia Questionnaire) and Their Associations with Distress among Malaysian University Students

**DOI:** 10.3390/healthcare10081448

**Published:** 2022-08-02

**Authors:** Serene En Hui Tung, Wan Ying Gan, Jung-Sheng Chen, Kamolthip Ruckwongpatr, Iqbal Pramukti, Siti R. Nadhiroh, Yen-Ling Chang, Chien-Chin Lin, Amir H. Pakpour, Chung-Ying Lin, Mark D. Griffiths

**Affiliations:** 1 Division of Nutrition and Dietetics, School of Health Sciences, International Medical University, Kuala Lumpur 57000, Malaysia; serenetung84@gmail.com; 2 Department of Nutrition, Faculty of Medicine and Health Sciences, Universiti Putra Malaysia, Serdang 43400, Malaysia; wanying@upm.edu.my; 3 Department of Medical Research, E-Da Hospital, Kaohsiung 82445, Taiwan; ed113187@edah.org.tw; 4 Institute of Allied Health Sciences, College of Medicine, National Cheng Kung University, Tainan 701401, Taiwan; kamolthip.681@gmail.com; 5 Department of Community Health Nursing, Faculty of Nursing, Universitas Padjadjaran, Bandung 45363, Indonesia; iqbalrevi@gmail.com; 6 Department of Nutrition, Faculty of Public Health, Universitas Airlangga, Surabaya 60115, Indonesia; sitinadhiroh@fkm.unair.ac.id; 7 Department of Family Medicine, Cardinal Tien Hospital, New Taipei 23148, Taiwan; 8 Department of Laboratory Medicine, National Taiwan University Hospital, Taipei 100225, Taiwan; jake9665@gmail.com; 9 Division of Hematology and Internal Medicine, National Taiwan University Hospital, Taipei 100225, Taiwan; 10 Graduate Institute of Clinical Medicine, National Taiwan University, Taipei 106319, Taiwan; 11 Department of Nursing, School of Health and Welfare, Jönköping University, SE-551 11 Jönköping, Sweden; amir.pakpour@ju.se; 12 Biostatistics Consulting Center, National Cheng Kung University Hospital, College of Medicine, National Cheng Kung University, Tainan 701401, Taiwan; 13 Department of Occupational Therapy, College of Medicine, National Cheng Kung University, Tainan 701401, Taiwan; 14 Department of Public Health, College of Medicine, National Cheng Kung University, Tainan 701401, Taiwan; 15 International Gaming Research Unit, Psychology Department, Nottingham Trent University, Nottingham NG1 4FQ, UK; mark.griffiths@ntu.ac.uk

**Keywords:** smartphone addiction, social media addiction, problematic internet use, psychometrics, university students, Rasch analysis

## Abstract

The present study evaluated the psychometric properties of four instruments related to internet use, namely the Bergen Social Media Addiction Scale (BSMAS), Smartphone Application-Based Addiction Scale (SABAS), (nine-item) Internet Gaming Disorder Scale Short Form (IGDS9-SF), and Nomophobia Questionnaire (NMPQ) as well as their associations with psychological distress among Malaysian university students. A total of 380 Malaysian university students (71.6% females, mean age 24.0 years) were recruited through convenience sampling and completed an online survey including questions concerning socio-demographic background, social media addiction, smartphone addiction, internet gaming disorder, and nomophobia. Confirmatory factor analysis and Rasch analysis were applied to evaluate the psychometric properties of the instruments and Cronbach’s alpha value and McDonald’s omega value were used to confirm the internal consistency reliability of the instruments. The unidimensional structure was confirmed for the BSMAS, SABAS, and IGDS9-SF while the four-factor structure was confirmed for NMPQ. All instruments showed good internal consistency reliability. Promising validity and reliability were confirmed for BSMAS, SABAS, IGDS9-SF, and NMPQ. Therefore, they are useful to assess different types of problematic internet use among university students in Malaysia. Furthermore, a significant association was observed between internet use and psychological distress. The present study is the first to investigate the validity and reliability of BSMAS, SABAS, IGDS9-SF, and NMPQ among Malaysian university students using rigorous psychometric testing methods (i.e., Rasch analysis).

## 1. Introduction

The advancement in technology has led to great use of the internet and young adults are major consumers of internet-based applications and platforms [1]. Because of its convenience, the internet has become an important part of individuals’ lives, and this was especially prevalent during the COVID-19 pandemic given the various restrictions forcing individuals to rely on digital technology more than ever before [2,3]. It is estimated that approximately 60% of the world’s population uses the internet and that Asia has the largest number of internet users followed by Europe [4].

In Malaysia (where the present study was carried out), the Department of Statistics Malaysia (in 2020) reported that 91.7% of Malaysian households had access to the internet via smartphones (98.2%) and computers (80.0%) and that the prevalence of internet use was 89.6%. Malaysian internet users were reported to use the internet for social networking use (98.0%) and playing or downloading games (87.9%) [5]. One of the reasons that internet use has become so prevalent is the global use of smartphones. The constant availability of smartphones with potentially addictive applications such as social networking sites and online video games can be problematic for a minority [6].

While many benefits have been proposed and confirmed for the use of the internet, such as communication, teaching and learning, information gathering, staying connected with friends and family, and entertainment, the lack of control regarding internet use has also led to an increase in detrimental health-related outcomes both physically and mentally [7]. Some of these health outcomes include poor sleep, musculoskeletal discomfort [8], and psychological distress, such as depression, anxiety, and stress [2,3]. These problems have been reported among social media users [9,10], those having problems with online gaming [11], and problematic smartphone users [12]. Moreover, a phenomenon of fear of not having a smartphone on hand has also been observed and has been termed ‘nomophobia’ (no mobile phobia). Similar to the aforementioned potentially addictive behaviors, nomophobia is related to negative mental health-related outcomes [13].

Although internet addiction is not classified as a mental disorder in the latest (fifth) edition of the *Diagnostic and Statistical Manual of Mental Disorders* (DSM-5), one online activity has been proposed as a tentative addictive disorder in the DSM-5 (i.e., internet gaming disorder) [14]. Given the importance of assessing various online addictive behavior uses among youth due to the various detrimental health outcomes, psychometric instruments to assess these potentially addictive behaviors are important to identify the severity. Although problematic internet use has been investigated in the past in Malaysia [15,16], these studies were conducted among children, medical students, and patients with depression. Therefore, there is still a lack of research conducted in understanding the addictive behaviors related to internet and smartphone use, especially among university students in Malaysia. As overdependence on the internet is prevalent among young people in Malaysia [16], there is a need to validate instruments that assess problematic use for prevalence profiles and prevent problematic internet use among Malaysian university students.

To date, there are few Malaysian studies to support the use of the Bergen Social Media Addiction Scale (BSMAS), Smartphone Application-Based Addiction Scale (SABAS), (nine-item) Internet Gaming Disorder Scale Short Form (IGDS9-SF), and Nomophobia Questionnaire (NMPQ) in Malaysia. More specifically, the psychometric evidence of these instruments used in Malaysia has not previously been examined through the application of Rasch analysis, a powerful statistical method that more robustly evaluates the validity and reliability of an instrument in comparison to the classical test theory [17]. Therefore, the purpose of the present study was to examine the psychometric properties of BSMAS, SABAS, IGDS9-SF, and NMPQ among Malaysian university students. After validating the BSMAS, SABAS, IGDS9-SF, and NMPQ, their associations with psychological distress were further investigated.

## 2. Materials and Methods

### 2.1. Participants and Data Collection

The participants of the study were recruited between 19 August 2021 and 30 September 2021 utilizing a convenience sampling method. The recruitment was conducted using online social media platforms including *Facebook*, *WhatsApp*, *Instagram*, and *Telegram* and hosted on *Google Forms*. The inclusion criteria of the present study were (i) being a university student and (ii) residing in Malaysia during the survey period. The exclusion criteria were university students who (i) were pregnant or lactating females; (ii) self-reported as having chronic diseases such as cardiovascular diseases and cancer; and (iii) were physically disabled. These exclusion criteria were used because they may influence an individual’s internet use and subsequently impact the answers to the internet-related instruments used in the present study. All participants were required to provide informed consent (provided on the first page of the online survey) by clicking the option “*I agree to participate in the study*” before completing the survey. The questions relating to the exclusion criteria were in the initial sociodemographic questions in the online survey and were answered after the participants had agreed to complete the survey. However, no participants were removed from the analysis as none of the participants said they were physically disabled, had a chronic illness, and/or were pregnant/lactating. Study approval was obtained from the Ethics Committee for Research Involving Human Subjects in Universiti Putra Malaysia (JKEUPM; Reference Number: JKEUPM-2021-455) before the commencement of the study.

### 2.2. Measures

The measures were all in English because English is the main medium of instruction in universities and colleges in Malaysia. In addition, Malaysia is a multicultural country where different languages are used by different ethnic populations (e.g., Malays, Chinese, and Indians) making English a more appropriate language to be used in the present setting. However, cultural bias was likely to exist, and all measures were checked for their cultural adaptations by the present authors using the international guidelines on patient-reported outcomes [18,19]. More specifically, the Malaysian authors checked that all the content descriptions in the measures could be fully understood by the Malaysian participants.

### 2.3. Socio-Demographic Information

The background information obtained from the participants included age, gender (male or female), ethnicity (Malay, Chinese, or other), study program level (undergraduate or postgraduate), and marital status (single, married, or other). In addition, information on the concerning number of daily hours spent on social media and gaming was also obtained.

### 2.4. Bergen Social Media Addiction Scale (BSMAS)

The Bergen Social Media Addiction Scale (BSMAS) was used to assess social media addiction and experience of using social media within the past week [20]. It contains six items rated on a five-point Likert scale ranging from 1 (*very rarely*) to 5 (*very often*), yielding a total score ranging from 6 to 30. A higher score on the BSMAS indicates a greater risk of addiction to social media. The BSMAS has been translated into several languages and has shown acceptable psychometric properties across studies. The scale has shown good internal consistency reliability with a Cronbach’s α of 0.88 [20].

### 2.5. Smartphone Application-Based Addiction Scale (SABAS)

The Smartphone Application-Based Addiction Scale (SABAS) was used to assess the risk of developing smartphone addiction within the past week [21]. It contains six items rated on a 6-point Likert scale from 1 (*strongly disagree*) to 6 (*strongly agree*), yielding a total score ranging from 6 to 36. A higher score on the SABAS indicates that an individual is more at risk of addiction to a smartphone application. The scale has shown good internal consistency reliability (α = 0.81) and promising concurrent validity [21].

### 2.6. Internet Gaming Disorder Scale-Short Form (IGDS9-SF)

The Internet Gaming Disorder Scale-Short form (IGDS9-SF) was used to assess the severity of internet gaming disorder in both online and/or offline gaming activities within the past week [22]. It contains nine items rated on a five-point Likert scale ranging from 1 (*never*) to 5 (*very often*), yielding a total score ranging from 9 to 45. A higher score on the IGDS9-SF indicates a higher risk of internet gaming disorder. The scale has shown high internal consistency reliability (α = 0.90) and promising concurrent validity [23].

### 2.7. Nomophobia Questionnaire (NMPQ)

The Nomophobia Questionnaire (NMP-Q) was used to assess the risk of developing fear and being out of mobile phone contact within the past week [24]. The NMP-Q contains 20 items distributed across four factors (i.e., not being able to communicate, losing connectedness, not being able to access information, and giving up convenience). All the items are rated using a seven-point Likert scale from 1 (*strongly disagree*) to 7 (*strongly agree*), yielding a total score ranging from 20 to 140. A higher score on the NMP-Q indicates that an individual suffers more from the fear of being unable to use their mobile phone. The scale has shown high internal consistency reliability (α = 0.70–0.91) and promising concurrent validity [25].

### 2.8. Depression, Anxiety, Stress Scale (DASS-21)

The Depression, Anxiety, Stress Scale (DASS-21) was used to assess the level of psychological distress [26]. It contains 21 items (seven items on each subscale of depression, anxiety, and stress) rated on a four-point Likert scale ranging from 0 (*not applied at all*) to 3 (*applied very much*), yielding a total score ranging from 0 to 21 for each subscale. A higher score on the DASS-21 indicates a higher level of psychological distress. The scale has shown high internal consistency reliability (α = 0.926) and adequate construct validity among Malaysian university students [27].

### 2.9. Data Analysis

Descriptive statistics were first carried out to understand (i) the participants’ characteristics and (ii) the item score distributions on all four psychometric instruments. Then, three sets of psychometric testing methods were applied to evaluate the properties of the four instruments: (i) internal consistency; (ii) confirmatory factor analysis (CFA); and (iii) Rasch analysis.

For internal consistency, Cronbach’s α and McDonald’s ω were used to assess how the items in each of the four instruments (and the subscales for the NMPQ) adhered to each other. An acceptable value for either Cronbach’s α or McDonald’s ω is 0.7 or above [28,29]. For CFA, a diagonally weighted least squares estimator was used to test the factor structures for the four instruments separately. More specifically, unidimensional structure was tested for the BSMAS, SABAS, and IGDS9-SF. A four-factor structure was tested for the NMPQ. Moreover, several fit indices, including a nonsignificant χ^2^ test, a comparative fit index (CFI) > 0.9, a Tucker-Lewis index (TLI) > 0.9, a root mean square error of approximation (RMSEA) < 0.08, and a standardized root mean square residual (SRMR) < 0.08 were used together to evaluate whether the tested factor structures (i.e., unidimensional structure for BSMAS, SABAS, and IGDS9-SF; four-factor structure for NMPQ) were supported [30,31]. Factor loadings derived from the CFA were expected to be higher than 0.4 [32]. For Rasch analysis, a partial credit model (PCM) was used to test whether the items for the BSMAS, SABAS, and IGDS9-SF; and the items for the subscales of the NMPQ fitted their latent underlying concepts. Again, a nonsignificant χ^2^ test was used to support the fit between the item and its latent concept. Outfit and infit mean square (MnSq) were also used to test the fit. More specifically, both outfit and infit MnSq between 0.5 and 1.5 indicate good fit, while a value smaller than 0.5 indicates overfit (i.e., redundancy in the latent concept) and a value larger than 1.5 indicates misfit (i.e., out of the latent concept) [33,34]. All the statistical analyses were carried out using *R* software: the *psych* package was utilized for internal consistency [35]; the *lavaan* package was utilized for CFA [36]; and the *eRm* package was utilized for the Rasch analyses [37]. Moreover, a sample size of at least 300 was needed for the present study based on the participant-item ‘rule of thumb’ ratio. More specifically, a ratio of 15:1 was utilized (i.e., 15 participants for every item on the instrument with the most items). Given that the NMPQ has 20 items, a sample size of 300 was the minimal requirement (i.e., 15 × 20 = 300).

Lastly, Pearson correlations were used to examine the associations between the BSMAS, SABAS, IGDS9-SF, and NMPQ scores with the three DASS-21 subscale scores (i.e., depression, anxiety, and stress). IBM SPSS 20.0 (IBM Incorp.: Armond, NY, USA) was used for testing the Pearson correlation.

## 3. Results

The participants (*N* = 380) were generally young adults (mean age = 24.04 years; SD age = 5.07) with the majority being female (*n* = 272; 71.6%). Slightly over half of the participants were ethnic Chinese (*n* = 200; 52.6%), followed by Malay (*n* = 117; 30.8%). The participants spent an average spent 4.5 h daily on social media (SD = 3.21) and 1.36 h daily on gaming (SD = 2.01). Detailed information on the participants’ characteristics is shown in Table 1.

The descriptive statistics and distributions of all the items (including BSMAS, SABAS, IGDS9-SF, and NMPQ) are presented in Table 2. All the items demonstrated normal distributions according to small values of skewness (ranging between −0.03 and 0.41 for BSMAS; between −0.47 and 0.27 for SABAS; between 0.40 and 1.61 for IGDS9-SF; between −0.33 and 0.43 for NMPQ) and kurtosis (ranging between −0.95 and −0.62 for BSMAS; between −0.90 and −0.38 for SABAS; between −1.07 and 1.59 for IGDS9-SF; between −1.24 and −0.85 for NMPQ).

The internal consistencies of all four instruments were very good to excellent (Cronbach’s α = 0.86 and McDonald’s ω = 0.91 for BSMAS; Cronbach’s α = 0.88 and McDonald’s ω = 0.93 for SABAS; Cronbach’s α = 0.95 and McDonald’s ω = 0.96 for IGDS9-SF; and Cronbach’s α = 0.88 to 0.95 and McDonald’s ω = 0.90 to 0.97 for NMPQ). Moreover, CFA results supported the unidimensionality for the BSMAS (*p*-value of χ^2^ = 0.11; CFI = 0.996; TLI = 0.994; RMSEA = 0.039; and SRMR = 0.045), SABAS (*p*-value of χ^2^ = 0.36; CFI = 0.999; TLI = 0.999; RMSEA = 0.016; and SRMR = 0.038), and IGDS9-SF (*p*-value of χ^2^ = 0.99; CFI = 1.000; TLI = 1.005; RMSEA = 0.000; and SRMR = 0.037). The CFA results supported the four-factor structure of the NMPQ (*p*-value of χ^2^ = 1.00; CFI = 1.000; TLI = 1.003; RMSEA = 0.000; and SRMR = 0.037). Moreover, all the factor loadings in the CFA were larger than 0.4 (Table 3).

Rasch analyses additionally showed the good properties of all the items for all four instruments. More specifically, nonsignificant χ^2^ tests together with outfit MnSq and infit MnSq between 0.5 and 1.5 for all the items were found for the BSMAS (outfit MnSq = 0.78 to 0.96; infit MnSq = 0.79 to 0.96), the SABAS (outfit MnSq = 0.67 to 1.12; infit MnSq = 0.68 to 1.13), and the NMPQ (outfit MnSq = 0.59 to 1.13; infit MnSq = 0.62 to 1.05). Only one item in the IGDS9-SF showed misfit (i.e., Item 8 “*Do you play in order to temporarily escape or relieve a negative mood (e.g., helplessness, guilt, anxiety)?*”), with a significant χ^2^ test (*p* < 0.001) and large MnSq (outfit MnSq = 1.87 and infit MnSq = 1.86). However, all other items in the IGDS9-SF fit with the Rasch model (Table 4).

Significant and moderate associations were observed between each psychological distress and the four instruments of BSMAS (r = 0.393 to 0.428; *p*-values < 0.001), SABAS (r = 0.359 to 0.373; *p*-values < 0.001), IGDS9-SF (r = 0.331 to 0.392; *p*-values < 0.001), and NMPQ (r = 0.295 to 0.339; *p*-values < 0.001) (Table 5).

## 4. Discussion

To the best of the present authors’ knowledge, the present study is the first to examine the psychometric properties of four internet problem-related instruments (i.e., BSMAS, SABAS, IGDS9-SF, and NMPQ) among university students in Malaysia utilizing both classical and modern test theories. The psychometric results demonstrated good factor structures for all four instruments and confirmed the unidimensional structure of the BSMAS, SABAS, IGDS9-SF, and the four-factor structure of the NMPQ. Such factor structure findings were consistent with previous studies [38,39,40,41,42,43]. In short, all instruments demonstrated robust psychometric properties among Malaysian university students.

### 4.1. BSMAS

The results for BSMAS specifically showed that the scale had a unidimensional structure with acceptable internal consistency indicating that the instrument is able to assess the use of problematic social media use among Malaysia university students across different ethnic groups. The unidimensional structure of the instrument was found to be similar to recent Korean [38] and Spanish [41] studies. With the rise in the use of social media among youth for social interaction and information gathering [44], there is a need to ensure that the instrument used to assess problematic social media use is valid and tested for its psychometric properties. With the confirmed validity and the very good psychometric properties of BSMAS using the Rasch model analysis approach in the present study, the findings here echo the Rasch analysis findings of the Persian BSMAS, both showing that all BSMAS items embedded in the unidimensional structure [45].

### 4.2. SABAS

The findings for SABAS also indicated a unidimensional structure with very good internal consistency on this scale, confirming the reliability of the instrument in assessing problematic smartphone application use among university students. The findings related to the unidimensional structure and reliability were similar to a previous study [39]. Moreover, the findings of Rasch analysis in the present study concur with a prior Persian SABAS validation study [46] (i.e., all SABAS items embedded in the unidimensional structure). With the rise in the use of smartphones as a means of communication [47], there is a need to assess the dependency of users on smartphones due to the growing incidence of problematic smartphone use. Previous studies conducted in Malaysia have used the Smartphone Addiction Scale (SAS), [48] but a limitation of using the SAS is that it does not specifically assess the activity engagement of smartphone applications although the SAS has good psychometric properties. Therefore, the findings of the present study provide an alternative option with the focus on smartphone applications for healthcare providers and researchers to examine smartphone addiction among university students in Malaysia.

### 4.3. IGDS9-SF

Similar to BSMAS and SABAS, very good internal consistency and a unidimensional structure were found in the present study for IGDS9-SF, confirming findings from previous studies conducted among Turkish [40] and Sri Lankan [41] populations. The unidimensional structure of IGDS9-SF was also confirmed in a recent systematic review by Poon et al. [49] which examined the psychometric properties of the instrument in 21 studies comprising 15 different language versions (i.e., English, European Portuguese, South American Portuguese, Spanish, Albanian, Italian, Turkish, Slovenian, Polish, Persian, Malay, Korean, and Chinese, with three sublanguages of traditional Chinese in Hong Kong, traditional Chinese in Taiwan, and simplified Chinese in mainland China). Similarly, the one-factor structure of the IGDS9-SF was also found for the Malay version of the instrument in a previous study [50]. However, the psychometric properties of the Malay version of the instrument in the previous study were only tested using classical test theory and did not incorporate any Rasch analysis.

Despite the unidimensional structure found for the IGDS9-SF, Item 8 (“*Do you play in order to temporarily escape or relieve a negative mood [e.g., helplessness, guilt, anxiety]?*”) which assesses behavior escape from negative mood through gaming was found to have psychometric problems with relatively high infit and outfit MnSq values. One of the possible explanations is that this item may be unable to differentiate between disordered and non-disordered gamers. One of the explanations is that relieving a negative mood could be a form of positive coping strategy rather than a loss of control [51]. Therefore, the behavior escape assessed by Item 8 might be practiced by both disordered and non-disordered gamers [52]. This was confirmed by the low accuracy and specificity in the ability of this item to differentiate between disordered and non-disordered gamers in a previous study [53].

### 4.4. NMPQ

Lastly, the findings for NMPQ confirmed the four-factor structure of the scale with an acceptable range of internal consistency for all four factors. These findings support previous findings among Persians [25], Greek [54], Portuguese [55], and Indonesian populations [56] where similar reliability and four-factor structure were observed in both populations. However, it should be noted that the previous Persian study was conducted among adolescents rather than university students. Nevertheless, findings from the present study confirm that the NMPQ is a valid and robust research instrument due to its good psychometric properties and internal consistencies. Therefore, the NMPQ is able to assess nomophobia among university students in Malaysia.

### 4.5. Associations between Psychological Distress and the Four Instruments

The findings from this study were consistent with previous studies [42,57,58,59], whereby BSMAS, SABAS, IGDS9-SF, and NMPQ were correlated with depression, anxiety, and stress. A systematic review looking into problematic social networking site use (PSNSU) assessed through BSMAS and comorbid psychiatric disorders in recent large-scale studies found positive associations between BSMAS and depression, anxiety, and stress [57]. Similarly, a study among a 964 sample of internet gamers recruited online found a positive relationship between anxiety, depression, and internet gaming assessed using the IGDS9-SF [58]. Another study among 11,014 primary and secondary schoolteachers examined the association between problematic internet use, psychological distress, and sleep problems during COVID-19 and found positive correlations between BSMAS and SABAS with psychological distress among both high sleep and low sleep problem groups [59]. As for nomophobia, similar results were found where a positive correlation was found between NMPQ with depression, anxiety, and stress among 500 European Portuguese adults [43]. The relationship between psychological distress and BSMAS was the strongest compared to SABAS, IGDS9-SF, and NMPQ which further confirms the conclusion that problematic social networking site use among adults is associated with comorbid psychiatric disorder outcomes [57].

### 4.6. Implications

Some of the practical implications of the present study include that these four instruments could be used to study problematic internet use behaviors among Malaysian university students by researchers and healthcare practitioners in the future. The risk of possible internet addiction could also be assessed through these instruments. Nevertheless, these instruments are non-diagnostic tools, and further development of clinical diagnostic criteria is needed to assess disordered internet use behaviors.

### 4.7. Limitations

There are a number of study limitations. First, the instruments were only tested among university students making it difficult to generalize the results to other age groups such as children and adults. Therefore, there is a need to explore the psychometric properties of these instruments among individuals across the lifespan and of other age groups. Second, the participants were recruited using a convenience sampling method, meaning the results are not generalizable to the whole population of university students in Malaysia. Third, the self-reported nature of the instruments could lead to biased findings due to participants providing socially desirable answers and experiencing recall bias. Fourth, as mentioned in Section 4.6, these instruments are non-diagnostic tools and cannot be used for diagnosis. Moreover, some of these behaviors are likely to be a consequence of other comorbid disorders. Therefore, future studies are needed to examine the association between problematic internet use and comorbid psychiatric disorders, which would likely expand the usefulness of these instruments. Fifth, the present sample had a gender imbalance (i.e., 72% female participants). Two reasons may explain the high ratio of female participants in the present study: (i) the proportion of female students in Malaysian public higher education institutions has been approximately 60% since 2012 [60]; (ii) females are more likely than males to participate in scientific studies [61]. Therefore, future psychometric evaluations of these instruments need to have more gender-balanced samples to corroborate the present study’s findings. Sixth, the present sample was homogenous (given that all the participants were university students and predominantly female). Therefore, it is not known if the instruments evaluated in the present study can be applied to non-university students with diverse characteristics. Consequently, it is recommended that future studies should recruit more diverse samples (i.e., participants with a much broader demographic of education, age, gender, socioeconomic status, ethnicity, marital status, and type of job (e.g., knowledge worker versus manual laborer)) to further evaluate the psychometric properties of these instruments and their relationships with mental health. Given this limitation, the present study was unable to determine if Malaysian university students had higher levels of problematic internet use than non-university students.

## 5. Conclusions

The findings of the present study demonstrated that BSMAS, SABAS, IGDS9-SF, and NMPQ are valid and reliable instruments and are easy to use in assessing internet problem-related behaviors among university students in Malaysia. In addition, the psychometric properties of these instruments were found to be robust and valid. However, special attention is needed for Item 8 of IGDS9-SF (i.e., using gaming to escape) due to the misfit in the Rasch model. Furthermore, a significant association was found between internet use and psychological distress, which further confirms the concerns about internet use and addiction among university students.

## Figures and Tables

**Table 1:** Characteristics of the participants (*N* = 380).

	*n* (%) or M (SD)
Age in year	24.04 (5.07)
Gender	
*Male*	108 (28.4)
*Female*	272 (71.6)
Ethnicity group	
*Malay*	117 (30.8)
*Chinese*	200 (52.6)
*Indian*	39 (10.3)
*Others*	24 (6.3)
Study program	
*Undergraduate*	277 (72.9)
*Postgraduate*	103 (27.1)
Marital status	
*Single*	350 (92.1)
*Married*	28 (7.4)
*Other*	2 (0.5)
Daily hours on social media	4.50 (3.21)
Daily hours on gaming	1.36 (2.01)

**Table 2:** Score distributions for the items of the internet-related instruments.

	Mean	SD	Skewness	Kurtosis	Range
BSMAS	16.80	5.40	0.17	−0.45	6–30
Item B1	2.93	1.10	−0.03	−0.62	1–5
Item B2	3.03	1.14	0.01	−0.72	1–5
Item B3	2.93	1.25	−0.01	−0.95	1–5
Item B4	2.87	1.13	0.09	−0.67	1–5
Item B5	2.52	1.16	0.34	−0.75	1–5
Item B6	2.51	1.21	0.41	−0.75	1–5
SABAS	21.44	6.71	−0.06	−0.53	6–36
Item S1	3.96	1.41	−0.43	−0.73	1–6
Item S2	3.19	1.38	0.14	−0.76	1–6
Item S3	3.89	1.41	−0.46	−0.58	1–6
Item S4	4.00	1.34	−0.47	−0.38	1–6
Item S5	3.16	1.43	0.17	−0.85	1–6
Item S6	3.24	1.48	0.27	−0.90	1–6
IGDS9-SF	16.87	8.26	0.94	0.09	9–43
Item I1	2.05	1.12	0.76	−0.43	1–5
Item I2	1.77	1.04	1.27	0.86	1–5
Item I3	1.93	1.15	1.03	−0.01	1–5
Item I4	1.86	1.06	1.03	0.10	1–5
Item I5	1.74	1.06	1.33	0.88	1–5
Item I6	1.81	1.09	1.24	0.55	1–5
Item I7	1.68	1.04	1.46	1.23	1–5
Item I8	2.48	1.35	0.40	−1.07	1–5
Item I9	1.56	0.95	1.61	1.59	1–5
NMPQ	78.71	28.67	−0.003	−0.80	20–140
NMPQ_F1	17.05	6.28	−0.25	−0.82	4–28
Item N1	4.23	1.79	−0.19	−0.95	1–7
Item N2	4.53	1.74	−0.29	−0.89	1–7
Item N3	3.87	1.85	−0.08	−1.12	1–7
Item N4	4.42	1.75	−0.30	−0.85	1–7
NMPQ_F2	19.42	7.79	−0.04	−0.83	5–35
Item N5	3.88	1.94	0.07	−1.18	1–7
Item N6	3.47	1.88	0.26	−1.03	1–7
Item N7	4.29	1.93	−0.21	−1.12	1–7
Item N8	3.89	1.91	0.01	−1.13	1–7
Item N9	3.89	1.86	0.00	−1.09	1–7
NMPQ_F3	24.63	9.97	−0.12	−1.00	6–42
Item N10	3.81	1.93	0.08	−1.18	1–7
Item N11	4.47	1.87	−0.33	−1.00	1–7
Item N12	4.11	1.87	−0.14	−1.09	1–7
Item N13	4.29	1.85	−0.21	−1.05	1–7
Item N14	4.17	1.82	−0.18	−1.00	1–7
Item N15	3.79	1.86	0.05	−1.16	1–7
NMPQ_F4	17.60	8.11	0.21	−0.91	5–35
Item N16	3.22	1.88	0.43	−0.99	1–7
Item N17	3.43	1.83	0.26	−1.05	1–7
Item N18	3.58	1.89	0.15	−1.13	1–7
Item N19	3.62	1.87	0.13	−1.12	1–7
Item N20	3.75	1.97	0.09	−1.24	1–7

BSMAS = Bergen Social Media Addiction Scale; SABAS = Smartphone Application Based Addiction Scale; IGDS9-SF = [Nine-item] Internet Gaming Disorder Scale-Short Form; NMPQ = Nomophobia Questionnaire; NMPQ_F1 = Not being able to access information; NMPQ_F2 = Giving up convenience; NMPQ_F3 = Not being able to communicate; NMPQ_F4 = Losing connectedness.

**Table 3:** Psychometric results derived from confirmatory factor analysis on the internet-related instruments.

	BSMAS	SABAS	IGDS9-SF	NMPQ			
Factor loading				NMPQ_F1 ^a^	NMPQ_F2 ^a^	NMPQ_F3 ^a^	NMPQ_F4 ^a^
Item 1	0.71	0.64	0.84	0.81	0.72	0.87	0.83
Item 2	0.76	0.75	0.87	0.82	0.73	0.84	0.86
Item 3	0.73	0.80	0.86	0.87	0.77	0.89	0.83
Item 4	0.70	0.81	0.88	0.84	0.83	0.86	0.77
Item 5	0.74	0.74	0.81	--	0.78	0.89	0.83
Item 6	0.68	0.74	0.89	--	--	0.85	--
Item 7	--	--	0.80	--	--	--	--
Item 8	--	--	0.66	--	--	--	--
Item 9	--	--	0.76	--	--	--	--
Fit indices							
χ^2^ (df)	14.23 (9)	9.92 (9)	13.17 (27)	107.92 (164)			
*p*-value	0.11	0.36	0.99	1.00			
CFI	0.996	0.999	1.000	1.000			
TLI	0.994	0.999	1.005	1.003			
RMSEA	0.039	0.016	0.000	0.000			
SRMR	0.045	0.038	0.037	0.037			

BSMAS = Bergen Social Media Addiction Scale; SABAS = Smartphone Application Based Addiction Scale; IGDS9-SF = [Nine-item] Internet Gaming Disorder Scale-Short Form; NMPQ = Nomophobia Questionnaire; NMPQ_F1 = Not being able to access information; NMPQ_F2 = Giving up convenience; NMPQ_F3 = Not being able to communicate; NMPQ_F4 = Losing connectedness; CFI = comparative fit index; TLI = Tucker-Lewis index; RMSEA = root mean square error of approximation; SRMR = standardized root mean square residual. ^a^ The items depended on the item number for that specific factor; for example, Item 2 for NMPQ_F2 is the fifth item for the entire NMPQ.

**Table 4:** Psychometric results derived from Rasch analysis on the internet-related instruments.

	χ^2^ (df)	*p*-Value	Outfit MnSq	Infit MnSq
BSMAS				
Item B1	318.94 (362)	0.95	0.88	0.88
Item B2	284.03 (362)	1.00	0.78	0.79
Item B3	315.25 (362)	0.96	0.87	0.83
Item B4	328.67 (362)	0.90	0.91	0.90
Item B5	299.58 (362)	0.99	0.83	0.81
Item B6	348.39 (362)	0.69	0.96	0.96
SABAS				
Item S1	412.55 (369)	0.06	1.12	1.13
Item S2	308.79 (369)	0.99	0.84	0.83
Item S3	269.52 (369)	1.00	0.73	0.73
Item S4	246.98 (369)	1.00	0.67	0.68
Item S5	331.30 (369)	0.92	0.90	0.88
Item S6	329.14 (369)	0.93	0.89	0.90
IGDS9-SF				
Item I1	260.66 (272)	0.68	0.96	0.91
Item I2	160.46 (272)	1.00	0.59	0.68
Item I3	201.62 (272)	1.00	0.74	0.80
Item I4	181.27 (272)	1.00	0.66	0.70
Item I5	233.58 (272)	0.96	0.86	0.90
Item I6	143.75 (272)	1.00	0.53	0.61
Item I7	209.63 (272)	1.00	0.77	0.84
Item I8	511.56 (272)	<0.001	1.87	1.86
Item I9	195.90 (272)	1.00	0.72	0.88
NMPQ				
NMPQ_F1				
Item N1	300.34 (358)	0.99	0.84	0.84
Item N2	226.97 (358)	1.00	0.63	0.62
Item N3	319.70 (358)	0.93	0.89	0.86
Item N4	264.56 (358)	1.00	0.74	0.76
NMPQ_F2				
Item N5	309.39 (355)	0.96	0.87	0.92
Item N6	273.10 (355)	1.00	0.77	0.81
Item N7	286.26 (355)	1.00	0.80	0.81
Item N8	262.99 (355)	1.00	0.74	0.75
Item N9	320.29 (355)	0.91	0.90	0.91
NMPQ_F3				
Item N10	372.04 (347)	0.17	1.07	1.05
Item N11	267.68 (347)	1.00	0.77	0.81
Item N12	275.19 (347)	1.00	0.79	0.79
Item N13	209.69 (347)	1.00	0.60	0.63
Item N14	296.00 (347)	0.98	0.85	0.83
Item N15	360.07 (347)	0.30	1.04	1.00
NMPQ_F4				
Item N16	294.66 (340)	0.96	0.86	0.87
Item N17	222.45 (340)	1.00	0.65	0.65
Item N18	201.80 (340)	1.00	0.59	0.62
Item N19	384.68 (340)	0.048	1.13	1.05
Item N20	329.46 (340)	0.65	0.97	0.95

BSMAS = Bergen Social Media Addiction Scale; SABAS = Smartphone Application Based Addiction Scale; IGDS9-SF = [Nine-item] Internet Gaming Disorder Scale-Short Form; NMPQ = Nomophobia Questionnaire; NMPQ_F1 = Not being able to access information; NMPQ_F2 = Giving up convenience; NMPQ_F3 = Not being able to communicate; NMPQ_F4 = Losing connectedness; Outfit MnSq = outfit mean square; Infit MnSq = infit mean square.

**Table 5:** Correlation between psychological distress and internet-related instruments.

		*r*	
	Depression	Anxiety	Stress
BSMAS	0.428	0.393	0.421
SABAS	0.368	0.359	0.373
IGDS9-SF	0.392	0.331	0.357
NMPQ	0.304	0.295	0.339

BSMAS = Bergen Social Media Addiction Scale; SABAS = Smartphone Application Based Addiction Scale; IGDS9-SF = (Nine-item) Internet Gaming Disorder Scale-Short Form; NMPQ = Nomophobia Questionnaire. Note: All *p*-values < 0.001.

## Data Availability

The datasets generated during and/or analyzed during the current study are available from the corresponding author on reasonable request.
